# Development and validation of the HIV adolescent readiness for transition scale (HARTS) in South Africa

**DOI:** 10.1002/jia2.25767

**Published:** 2021-07-08

**Authors:** Brian C Zanoni, Moherndran Archary, Thobekile Sibaya, Nicholas Musinguzi, Mary E Kelley, Shauna McManus, Jessica E Haberer

**Affiliations:** ^1^ Emory University Atlanta GA USA; ^2^ Children’s Healthcare of Atlanta Atlanta GA USA; ^3^ University of KwaZulu‐Natal Nelson Mandela School of Medicine Durban South Africa; ^4^ King Edward VIII Hospital Durban South Africa; ^5^ Global Health Collaborative University of Science and Technology Mbarara Uganda; ^6^ Rollins School of Public health Atlanta GA USA; ^7^ Massachusetts General Hospital Boston MA USA; ^8^ Harvard Medical School Boston MA USA

**Keywords:** adolescent, HIV, South Africa, healthcare transition readiness, scale development

## Abstract

**Introduction:**

Adolescents living with perinatally acquired HIV have low rates of retention in care and viral suppression after the transition from paediatric to adult care. In this study, we developed and validated a tool to identify adolescent transition readiness.

**Methods:**

We developed the HIV Adolescent Readiness for Transition Scale (HARTS) from June 2016 to May 2019 by iteratively adapting existing transition readiness scales for other chronic illnesses by conducting focus groups with 11 healthcare providers and 20 adolescents in South Africa. We administered a preliminary questionnaire to 131 adolescents to determine psychometric properties and assess test–retest variability. We used confirmatory factor analysis to verify the proposed scale structure using the underlying variable approach. We correlated responses to self‐described transition readiness and age using linear regression. We subsequently validated the scale by prospectively administering it to 199 adolescents in a second South African setting before their transition. We then used multivariable logistic regression to assess the effects of the HARTS and relevant socio‐behavioural covariates on viral suppression one year after transition.

**Results:**

We identified four domains relevant to transition readiness: disclosure, health navigation, self‐advocacy and health literacy. Fifteen questions with a significant factor loading of 0.3 to 0.9 were identified. No significant test–retest variability was seen among 10% of participants. Positive correlations with self‐described transition readiness were significant with the overall HARTS and domains of health navigation, self‐advocacy and health literacy. In the prospective analysis, for adolescents not using drugs, each 10‐point increase in the HARTS was associated with 0.62 odds of viral failure (95% CI 0.45 to 0.86; *p* = 0.004). The individual domains of self‐advocacy (AOR 0.56; 95% CI 0.33 to 0.94; *p* = 0.029), disclosure (AOR 0.02; 95% CI 0.01 to 0.25; *p* = 0.002), health navigation (AOR 0.51; 95%CI 0.25 to 1.02; *p* = 0.056) and health literacy (AOR 0.37; 95% CI 0.10 to 1.30; *p* = 0.121) were associated with viral failure adjusting for age at antiretroviral therapy initiation, ART regimen, sex, disclosure status, and alcohol use in both analyses.

**Conclusions:**

The HARTS is a validated scale that can be used to identify which adolescents may require additional interventions prior to transitioning to adult care to improve viral suppression after transition.

## 1 Introduction

Despite improved access to HIV diagnosis and antiretroviral therapy (ART) in recent years, HIV remains the leading cause of death among adolescents in South Africa where less than 50% of adolescents living with HIV are virally suppressed [1, 2, 3, 4]. One major area of vulnerability is the transition from paediatric to adult‐based care for adolescents living with HIV. Guidelines about the transition process are unclear about the optimal age, timing or preparation for adolescents, caregivers or their healthcare providers [5, 6, 7]. The timing of transition to adult care is often arbitrarily assigned based on age and commonly occurs with little or no preparation resulting in adolescents transitioning between the ages of 12 and 24 years. The lack of clear evidence‐based transition protocols may contribute to the high rates of loss to follow‐up, virological failure and death among adolescents living with perinatally acquired HIV (ALPH) after transitioning to adult care [2, 8, 9, 10].

Several scales have been developed to evaluate healthcare transition readiness for adolescents with chronic illness as they transition from paediatric care to adult care. These scales were developed in North America for children with chronic illnesses and may not be appropriate for ALPH in South Africa [11, 12, 13]. These generic scales do not account for complex social and behavioural concepts specific to HIV, such as stigma and HIV disclosure. Generic transition readiness assessments have also not been validated based on clinical outcomes. They typically report effects based on age and self‐described transition readiness, limiting their utility in predicting successful transition to adult care [11, 12].

To fill this gap in the literature, we designed and validated the HIV Adolescent Readiness to Transition Scale (HARTS) questionnaire to help determine when an adolescent is ready to transition to adult care and to identify which adolescents may need further interventions prior to transition to improve viral suppression rates after transition.

## 2 Methods

### 2.1 Study design overview

**Figure 1:**
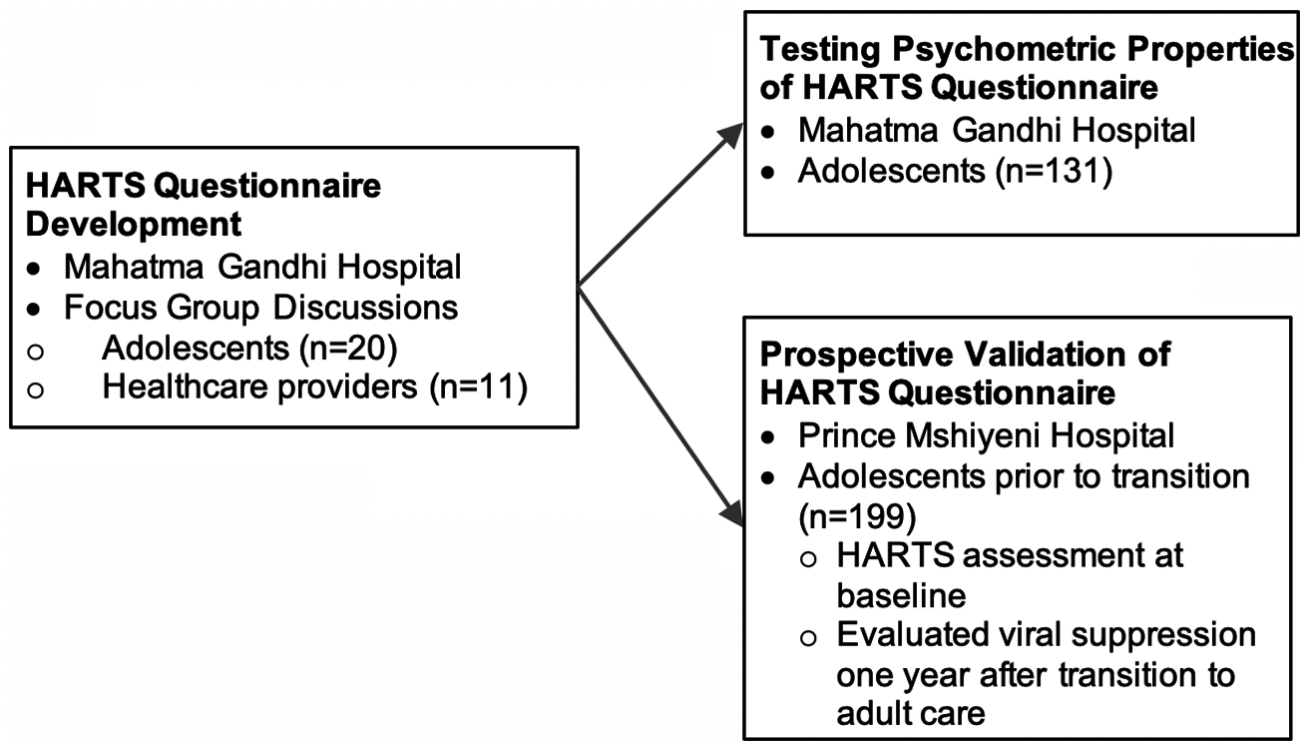
Study design.

The preliminary HARTS questionnaire was developed based on existing general healthcare transition readiness assessments (TRAQ and TRANSITION Q) from the literature for children with chronic illnesses in North America [11, 13, 14]. As part of formative development, the questionnaire was iteratively modified based on focus group discussions with adolescents and healthcare providers. Questions were removed and new domains/questions were added based on participant feedback. We then reviewed the preliminary HARTS questionnaire with additional focus groups and obtained feedback on the questionnaire instructions, design and content, including wording, language, ease of use and generalizability. We then assessed readability for the final HARTS questionnaire by calculating the Flesch–Kincaid score (Phase 1). It was next administered to adolescents prior to transitioning to adult care to measure the questionnaire’s psychometric properties (Phase 2). Subsequently, the questionnaire was prospectively validated in a separate cohort of adolescents by administering it prior to transition during their last visit in the paediatric clinic and measuring viral suppression (viral load < 200 copies/mL) one year after transition to adult care as indicated in Figure 1 (Phase 3).

### 2.2 Phase 1 – Scale development

#### 2.2.1 Setting

Mahatma Gandhi Memorial Hospital is a regional/district hospital located in the township of KwaMashu outside of Durban, South Africa. The outpatient paediatric clinic provides care for more than 650 children less than age 15 who are receiving ART. Transition to adult care occurs when adolescents meet the following criteria: age >15 years, disclosed of their HIV status, and taking adult doses of antiretroviral therapy. This may occur with little prior preparation or discussion.

#### 2.2.2 Recruitment and consent

The formative questionnaire development took place between June 2016 and August 2017. Adolescent study participants (n = 20) were recruited by convenience sampling during their routine clinic visits prior to transitioning to adult clinic. They were between 15 and 24 years old, acquired HIV perinatally, aware of their HIV status and receiving ART. Adolescents with developmental delay interfering with the consent procedures were excluded from participation. Adolescents less than 18 years old assented to study participation and written consent was obtained from the primary caregiver. Adolescents 18 years or older provided their own informed consent.

Additionally, we recruited a convenience sample of healthcare providers (n = 11) (physicians, nurses and counsellors) involved in adolescent HIV care and treatment at Mahatma Gandhi Memorial Hospital. Healthcare providers all provided written informed consent.

#### 2.2.3 Formative development

We used open‐ended questions during focus groups to obtain information on potentially important factors necessary for transition readiness for ALPH in South Africa. Two focus groups with adolescents (n = 20) and three focus groups with healthcare providers (n = 11) were held separately each lasting approximately 90 minutes. Topics in the interview guide included HIV knowledge; experience with clinic providers; structural barriers and facilitators to adherence and clinic attendance; experience with HIV status disclosure and stigma; social support from peers, family and community; facilitators and barriers to transition and self‐perceived transition readiness. We also reviewed published healthcare transition readiness scales (TRAQ and TRANSITION R) and transition models (GotTransition) based in North America for possible inclusion [11, 13, 15]. All focus groups were conducted by a female, bachelor’s‐level, research assistant trained in qualitative methods (author TS) who was not affiliated with the clinic. Focus groups were audio‐recorded, lasted between 60 and 90 minutes, and were conducted in isiZulu or English based on participant preference. Focus group content was transcribed verbatim and translated into English as needed by the research assistant who is bilingual in isiZulu and English.

#### 2.2.4 Analysis

We used an inductive content analysis approach to analyse qualitative data that was derived from reviewing, coding and interpreting the data [16, 17, 18]. Two researchers (BCZ and TS) reviewed the transcripts and analysed content to develop labels, create operational definitions and develop a codebook with selected illustrative quotes. The codebook was then refined using an iterative process using consensus through discussing discrepancies in coding until an agreed definition was reached. Using the coded data, themes were identified corresponding to important topics in the data. Theme examination included a combination of *a priori* categories (i.e. barriers to transition, facilitators to transition) and additional themes that emerged from the data. The themes were further organized into major domains and evidence was provided using illustrative quotes from the research participants.

### 2.3 Phase 2 – Psychometric property testing

#### 2.3.1 Recruitment and consent

To test the psychometric proprieties of the scale, we enrolled a separate cohort of adolescents (n = 131) who were living with HIV and receiving ART at Mahatma Gandhi Memorial Hospital to complete the preliminary HARTS questionnaire. We enrolled a convenience sample of adolescents using the same inclusion and exclusion criteria as described above. We used the same consent procedure as the formative scale development. For the psychometric testing, adolescents were enrolled from August 2017 to May 2019.

#### 2.3.2 Analysis

For the HARTS scale data, we needed to use a multi‐factor approach suitable for ordinal data to perform a confirmatory factor analysis (CFA) to verify the proposed scale structure as specified. Because IRT models are unidimensional by definition and expanding to multiple factors is for the most part computationally infeasible without very large sample sizes, we used the underlying variable (UV) approach [19, 20]. This approach assumes that each ordinal item is generated by an underlying unobserved variable that is normally distributed, with thresholds defining the categories c = 0, 1, 2, 3. The estimation proceeds by using the observed proportions in each category to estimate the threshold parameters that are then used to determine the polychoric correlation between each pair of variables using the assumption that the pair of underlying variables have a bivariate normal distribution. The set of univariate thresholds and the polychoric correlation matrix are then used to estimate the asymptotic covariance matrix, which is used as the weight matrix in an adaptation of a general family of weighted least squares (WLS) fit functions [20, 21] to obtain model estimates. This analysis was performed in LISREL 8.8. The comparative fit index (CFI) and the root mean squared error of approximation (RMSEA) were used to assess overall model fit.

We correlated the individual domains (disclosure, health literacy, healthcare navigation, self‐advocacy) and overall HARTS score to self‐described transition readiness and age using linear regression. We calculated Cronbach’s alpha with final HARTS questionnaire. We assessed test–retest variability with a convenience sample of 10% of the adolescents who took the questionnaire twice separated by approximately 3 hours and evaluated for the difference in mean overall score.

### 2.4 Phase 3 – Scale validation

#### 2.4.1 Setting

Prince Mshiyeni Hospital is a regional/district hospital located in the township of Umlazi outside of Durban, South Africa. The outpatient paediatric HIV clinic provides treatment for more than 800 children and adolescents living with HIV. In this Department of Health clinic, adolescents typically transition to adult care after they turn 12 years old without prior healthcare transition readiness assessment.

#### 2.4.2 Participant selection

We enrolled a convenience sample of ALPH who were fully aware of their HIV status, receiving ART (because the outcome measure was viral suppression), age > 12 years old (reflecting the age of transition in the clinic), prior to their transition to adult care during their last visit in the paediatric clinic (n = 199). Adolescents with developmental delay interfering with the consent procedures were excluded from participation.

#### 2.4.3 Recruitment

Using the same consenting procedures as above, study participants were enrolled from the paediatric clinic of Prince Mshiyeni Hospital during their routine clinic visits between August 2017 and May 2019.

#### 2.4.4 Data collection

The participants provided basic demographic information and completed a questionnaire prior to transitioning to adult clinic on their last day in the paediatric clinic. Measures in the questionnaire included: transition readiness (HARTS questionnaire), illicit drug and alcohol use (Youth Behavior Risk Survey) [22]; peer support (Adolescent Social Support Scale) [23] and self‐esteem (Rosenberg Self‐Esteem Scale) [24]. They were then followed for 12 months after transition to adult care. Viral suppression (<200 copies/mL) was evaluated 12 months after transition to adult clinical care through chart review of routine clinical samples.

#### 2.4.5 Analysis

Results were adjusted for potential covariates using bivariate analysis including age at ART initiation; ART regimen; biological sex; HIV disclosure status (as documented in the medical record by the healthcare team); illicit drug and alcohol use. Viral suppression was evaluated using multivariable logistic regression with Huber‐White robust standard errors, based on the continuous total value of the HARTS questionnaire and covariates with a *p* < 0.2 on bivariate analysis. In the multivariable regression, we also assessed for interaction between the HARTS score and each covariate and retained any interaction terms whose *p*‐value was <0.05. All statistical analyses were performed using Stata 13 (StataCorp, College Station, TX, USA).

For potential simplified prospective scale use, we dichotomized the total HARTS score to identify those adolescents who would most likely be ready to transition to adult care. In defining the binary threshold, we included the value that yielded the most correctly classified individuals identified by the combination of sensitivity and specificity of the continuous HARTS score in discriminating between viral failure and non‐failure after transitioning to adult care. We then assessed the sensitivity and specificity of this binary threshold in discriminating who was likely or not likely to have viral failure one year after transition. Sensitivity was computed as the proportion of individuals with viral failure at a given HARTS threshold, whereas specificity was the proportion without viral failure at that threshold.

The Durban University of Technology Institutional Research Ethics Committee, Biomedical Research Ethic Counsel of the University of KwaZulu‐Natal, KwaZulu‐Natal Department of Health, Partners Healthcare/Massachusetts General Hospital Research Ethics Board and the Emory University Institutional Review Board approved this protocol.

## 3 Results

### 3.1 Scale development

#### 3.1.1 Phase 1 – Formative development

We enrolled 20 adolescents (11 females, 9 males) prior to transition to adult care and 11 healthcare providers into focus group discussions. Healthcare providers included one doctor, four professional nurses, two nursing assistants, one enrolled nurse and three HIV counsellors. We identified four domains that were important to HIV transition readiness: disclosure, health navigation, self‐advocacy and health literacy as evidenced by selected illustrative quotes in Table 1. After completion of the iterative development process (see Supplement S1), the HARTS questionnaire included 16 questions as indicated in Table 2. Responses were coded on a 5‐point Likert scale (1. No; 2. No, but I am learning; 3. Yes, a little bit; 4. Yes, almost always, 5. Yes, always) and had a Flesch–Kincaid grade level of 6.3, indicating good readability.

**Table 1:** Domains determined by focus groups to be involved in healthcare transition readiness along with representative quotes

Domain	Participant	Selected quote
Disclosure	Healthcare provider	“It’s a process. It’s a systematic way to find out how much the child knows about HIV. Assess their knowledge, and then if the child does not know anything about HIV, leave it. Then slowly you can start again and again”
	Healthcare provider	Prior to transition, the clinic needs to “improve on disclosure tactics. Because right now, we are keeping 14‐year‐olds who are partially disclosed to. They should be transitioned”
	Adolescent prior to transition	“It’s important not to hold things inside, to talk about it, but those people that you trust are not just anyone. The people you trust about what you are going to tell them”
	Adolescent prior to transition	After disclosure “You feel that low self‐esteem. Even at school you can’t concentrate. You feel like you are different from others. Your life is controlled by the pills”
Healthcare navigation	Healthcare provider	“Some of them came in as babies. When they are old, suddenly, they must move now. They’ve never seen the nurses; they’ve never seen the doctors. Slowly we must introduce them to the nurses before we just throw them in the fire. So, the next visit is adults. They’ve never seen the doctors. They don’t even know who is in charge them”
	Adolescent prior to transition	“I understand the system better in paediatrics. On the adult side, I have to learn a whole new system”
Self‐advocacy	Healthcare provider	“If they take the medication by themselves and the results are Okay, the virus is suppressed. It means that person is taking those pills by themselves before they [transition]”
	Adolescent prior to transition	“I now must be responsible for myself and make choices for myself… You need someone, like yourself, self‐motivation to inspire you to do that thing”
	Adolescent prior to transition	“Before I [transition], I must be able to tell the doctors I have this problem, so they know how to deal”
Health literacy	Healthcare provider	“I always teach them and show them the viral load and the CD4. I encourage them that if they are taking their medication their viral load needs to be like that. Lower than that. And the CD4 needs to go up”
	Adolescent prior to transition	“It will be okay to move to the [adult clinic] because I will be learning more and different things”

**Table 2:** Standardized loadings for confirmatory factor analysis of the HARTS domains and overall HARTS score

HARTS question	HARTS domains			
	Disclosure	Health navigation	Self‐advocacy	Health literacy
1. Do you know why you take your medication?	0.675			
2. Do you know the names of your medications?	0.608			
3. Can you explain your health needs to doctors, nurses and counsellors?			0.418	
4. Can you explain your medical history to doctors, nurses and counsellors?	0.347			
5. Have you told anyone why you are taking your medication?	Question dropped			
6. Do you collect your own medication from the clinic?		0.731		
7. Do you take your medication by yourself?		0.391		
8. Do you travel on your own to your appointment?		0.902		
9. Do you know your clinic dates?		0.632		
10. Do you talk to the doctors, nurses and counsellors on your own?			0.453	
11. Do you see the doctors or nurses on your own during an appointment?			0.939	
12. Do you ask the doctors, nurses and counsellors questions?			0.326	
13. Do you answer the doctors’, nurses’ and counsellors’ questions?			0.315	
14. Do you know what illnesses should make you contact the clinic?				0.330
15. Do you make decisions about your own health?				0.643
16. Do you know your last viral load results?				0.354
Overall transition readiness	0.464	0.976	1.000	0.535

Values indicate the model coefficient for each domain.

#### 3.1.2 Phase 2 – Psychometric properties

We enrolled 131 adolescent participants for the psychometric evaluation of the HARTS questionnaire. Baseline demographic characteristics of adolescent participants are indicated by Table 3. At enrolment, the median age of participants was 14 years (IQR 13 to 15) with a median age of seven years at the time of ART initiation (IQR 5 to 9).

**Table 3:** Characteristics of adolescent participants involved in the HARTS scale development and validation

	Scale development (n = 131)	Scale validation (n = 199)
Enrolment characteristics		
Recruitment site	Mahatma Gandhi Memorial Hospital	Prince Mshiyeni Hospital
Median age at enrolment (years) (IQR)	14 (13 to 15)	13 (12 to 13)
Median age at ART initiation (years) (IQR)	7 (5 to 9)	8 (5 to 9)
Female, n (%)	64 (49%)	98 (49%)
First‐line ART regimen, n (%)	111 (85%)	144 (72%)
Lifetime alcohol use, n, %^a^	n/a	70 (35%)
Illicit drug use, n (%)	35 (3%)	19 (10%)
Documented disclosure, n (%)^a^	n/a	89 (45%)
Self‐esteem median (IQR)^a^, ^b^	n/a	19 (16, 22)
Peer‐support median (IQR)^a^, ^c^	n/a	39 (30, 44)

Outcomes	(n = 50)	(n = 199)
Viral suppression after transition, n (%)	39/50 (78%)	112 (57%)

^a^ Characteristics were only evaluated in the validation sample

^b^ median score on Rosenberg Self‐Esteem Scale

^c^ median score on Adolescent Social Support Scale

The CFA of the full scale could not be fit with preliminary question 5, which was determined to be caused by a lack of correlation with the rest of the scale using continuous data methods. Thus, question 5 was removed from the scale for further analysis. The remaining 15 items were fit to a second‐order UV factor model with the four domains as indicated (Table 2) and an additional higher order domain representing the total score. After anchoring on the self‐advocacy factor (setting loading to one) due to limited variance, we were able to demonstrate an adequate solution to the proposed structure with support for the use of both the subscales and total score as validated measures of readiness. The underlying factors were fitted to be independent of each other, consistent with their derivation, with the exception of a substantial correlation between disclosure and health literacy (r = 0.752). The CFI was 0.93 and the RMSEA was 0.0885, indicating an adequate fit for the purposes of reproducing existing scale structure for these data.

For the test–retest variability, the mean score was 36.3 (standard deviation (SD) 7.3) on the first test and 36.9 (SD 6.7) on the second test with no statistical difference in the means (*p* = 0.69). Positive correlations with self‐described transition readiness were significant with the overall HARTS score (coef 2.2; 95% CI 1.1 to 3.4; *p* < 0.001) and domains of health navigation (coef 0.6; 95% CI 0.1 to 1.0; *p* = 0.011), self‐advocacy (coef 0.8; 95% CI 0.3 to 1.3; *p* = 0.002) and health literacy (coef 0.6; 95%CI 0.2 to 0.9; *p* = 0.001) but not HIV disclosure (coef 0.3; 95% CI −0.1 to 0.6; *p* = 0.112). None of the individual domains nor the overall HARTS score significantly correlated with age at the time of transition. Cronbach’s alpha for the final 15 question HARTS questionnaire was 0.78.

#### 3.1.3 Phase 3: scale validation

We enrolled 199 adolescent participants for the validation of the HARTS questionnaire. At enrolment, the median age of participants was 13 years (IQR 12 to 13) with a median age of eight years at the time of ART initiation (IQR 5 to 9) as indicated in Table 3. At the time of transition, 144 (72%) were on first‐line ART, 19 (10%) self‐reported use of drugs and 89 (45%) had clearly documented HIV disclosure in the chart. All 199 of these adolescents transitioned to adult care after completion of their questionnaire, of whom 122 (57%) achieved viral suppression one year after the transition.

In the validation cohort, the HARTS scores ranged from 2 to 56 with a median of 31 (IQR 21 to 39). Based on interaction terms, the effect of HARTS score was dependent on reported illicit drug use (i.e. non‐alcohol, non‐marijuana drug use) (Table 4); the analysis was therefore stratified based on this factor. Each 10‐point increase in overall HARTS score was associated with 0.62 odds of viral failure (95% CI 0.45 to 0.86; *p* = 0.004) for participants not using illicit drugs and 2.01 odds of viral failure (95% CI 0.68 to 5.86, *p* = 0.20) for those reporting illicit drug use. For the individual HARTS domains: self‐advocacy (AOR 0.56; 95% CI 0.33 to 0.94; *p* = 0.029); disclosure (AOR 0.02; 95% CI 0.01 to 0.25; *p* = 0.002); health navigation (AOR 0.51; 95%CI 0.25 to 1.02; *p* = 0.056) and health literacy (AOR 0.37; 95% CI 0.10 to 1.30; *p* = 0.121) were associated with viral failure one year after transitioning to adult care (see Supplement S2 for multivariable models). The multivariable models adjusted for age at ART initiation, ART regimen, biological sex, HIV disclosure status, alcohol use and drug use.

**Table 4:** Multivariable logistic regression evaluating virological failure one year after transition to adult care for adolescents living with perinatally acquired HIV at Prince Mshiyeni Hospital, Umlazi, South Africa

Covariate	AOR	*p*‐value	95% CI
Age at ART initiation (years)	1.23	0.004	1.07 to 1.42
Female	2.58	0.014	1.21 to 5.51
Alcohol use	3.43	0.003	1.51 to 7.80
Disclosure	0.36	0.015	0.16 to 0.82
Illicit drug use	0.05	0.123	0.01 to 2.22
First‐line ART	0.07	<0.001	0.02 to 0.24
HARTS score (10‐point increase) stratified by illicit drug use			
First‐line ART and NOT using illicit drugs	0.61	0.004	0.44 to 0.85
First‐line ART and using illicit drugs	2.16	0.148	0.76 to 6.11

### 3.2 Scale performance

When dichotomizing the HARTS score to reflect those who had been ready to transition (i.e. those who were virally suppressed 12 months after transition) and those not who had not been ready to transition (i.e. those with viral failure 12 months after transition) for all participants, we chose a HARTS cutoff point of 45 or greater. This value represented the top 15% of HARTS scores and was significantly associated with viral suppression (*p* = 0.01). Individuals scoring 45 or higher on the HARTS had a specificity of 90.2% (95% CI 83.1 to 95) in predicting viral suppression one year after transition to adult care. Other performance characteristics included sensitivity of 21.4% (95% CI 13.2 to 31.7), positive predictive value 62.1% (42.3% to 79.3%) and negative predictive value of 60.5% (52.6 to 67.9) for scores 45 and greater predicting viral suppression one year after transition to adult care.

## 4 Discussion

We present the development, psychometric properties and validation of the HARTS questionnaire to measure transition readiness among ALPH in South Africa. The questionnaire captures key elements of the readiness process, including disclosure, health navigation, self‐advocacy and health literacy. It has no significant test–retest variability and a high Cronbach’s alpha. The overall HARTS score correlated with viral suppression one year after transition to adult clinic in a population without apparent need for intensified preparation (i.e. adolescents without reported drug use). Viral suppression is the goal of antiretroviral therapy and encompasses retention in care and adherence. Adolescents scoring 45 or greater on the HARTS are likely ready to transition to adult care without additional interventions, whereas those scoring less than 45 may require additional time or interventions prior to transition. The specificity for the HARTS to predict viral suppression in adolescents scoring 45 or greater on the HARTS is greater than 90% indicating adolescents who are likely ready to transition to adult care. This questionnaire can be used for healthcare providers and researchers to assist with the determination of transition readiness for adolescents prior to transition to adult care. Adolescents who are not yet ready to transition may benefit from targeted interventions to improve their transition readiness based on scores of the individual HARTS domains.

Three of the major domains associated with transition readiness in our population – health navigation, self‐advocacy and health literacy – have been identified as important factors in transition readiness in other transition scales developed for non‐HIV chronic illnesses in North America [11, 12, 13, 15, 25]. However, disclosure is a unique domain that accounts for the potentially stigmatizing nature of perinatally acquired HIV and incorporates the social dynamic of learning about HIV status. Disclosure has been identified as an important component of ART adherence, retention in care and transition care for ALPH [26, 27, 28, 29]. Future research may determine if results of individual domain scores may be used by healthcare providers to guide targeted interventions for adolescents who are not yet ready to transition to adult care.

This is the first transition readiness assessment developed and validated specifically for ALPH in South Africa. Currently, there are no clear guidelines on how to best determine healthcare transition readiness for ALPH in South Africa [30, 31, 32]. The HARTS is rapid self‐administered 15‐question questionnaire that could be integrated into busy clinics to determine adolescent readiness to transition or adapted to other settings to assist in healthcare transition for ALPH.

Due to the structure of paediatric health services and the absence of transition guidelines, age is the most common factor to determine transition readiness for ALPH. The HARTS questionnaire predicted viral suppression one year after transition to adult care, whereas age at transition was not associated with viral suppression in our study. The HARTS questionnaire can be used as a determinate for transition readiness to assist healthcare providers in determining which adolescents can transition to adult care without additional time in the paediatric clinic or additional interventions.

This study has several limitations. The study was validated in a single population with a narrow age range (ages 12 to 15) for transition to adult care. Future studies will be needed to assess the HARTS among adolescents who transition at older ages and in other settings. Although the HARTS was designed specifically for adolescents living with HIV, we intentionally do not include the term HIV due to varying understanding of disclosure among the adolescents. We do include the key concepts of disclosure and health literacy, which are important for healthcare transition readiness. In addition, we evaluated one‐year viral suppression after transition to adult care. Several studies have found higher retention in care and viral suppression in the first year after transition compared to longer term outcomes [8, 33]. The HARTS questionnaire will therefore also need to be validated for longer term outcomes.

## 5 Conclusions

The HARTS questionnaire is a validated scale that can be used to assist in the determination of which adolescents may require additional interventions prior to transitioning to adult care to improve viral suppression. It is particularly useful for adolescents without clear concern about the transition process (i.e. those who are not using drugs) to predict who may not need additional services prior to transition from paediatric to adult care.

## Competing interests

The authors declare no conflict of interest.

## Authors’ contributions

Dr. Zanoni conceptualized and designed the study, performed the literature review, assisted with the analysis, drafted the initial manuscript, reviewed and revised the manuscript and approved the final manuscript as submitted. Ms. Sibaya performed the data collection, contributed to the drafting of the manuscript, critically reviewed the manuscript and approved the final manuscript as submitted. Mr. Musinguzi performed the phase 3 validation data analysis, assisted with the drafting of the manuscript, reviewed and revised the manuscript and approved the final manuscript as submitted. Ms. McManus performed the psychometric data analysis, assisted with the drafting of the manuscript, reviewed and revised the manuscript and approved the final manuscript as submitted. Dr. Kelley performed the psychometric data analysis, assisted with the drafting of the manuscript, reviewed and revised the manuscript and approved the final manuscript as submitted. Dr. Archary assisted with the conceptualization and design of the study, contributed to the data collection, and critically reviewed the manuscript and approved the final manuscript as submitted. Dr. Haberer assisted with the conceptualization and design of the study, contributed to the analysis plan, reviewed and revised the manuscript and approved the final manuscript as submitted.

## Funding

This work was supported via: Harvard Center for AIDS Research NIH/NIAID 5P30AI060354‐13 PI: Zanoni; NICHD R21HD092132 PI: Zanoni; K24MH114732 PI: Haberer.

## Supporting information


**Supplement S1.** Formative development of the HARTS Questionnaire.

**Supplement S2.** Validation of individual domains using multivariable models assessing viral failure one year after transition to adult care.
