# Supplementary material for: Development and validation of the HIV adolescent readiness for transition scale (HARTS) in South Africa
Source: J Int AIDS Soc. 2021 Jul 8;24(7):e25767. doi: 10.1002/jia2.25767 (PMC8264413; doi:10.1002/jia2.25767)
Supplement: Supplementary file 1 — Supplement S1. Formative development of the HARTS Questionnaire. [file JIA2-24-e25767-s001.docx]

**Supplement 1: Formative Development of the HARTS Questionnaire:**

**Supplementary Table S1:** Demographics of adolescents participating in the formative development of the HARTS:

| Characteristics | n / (%) Total N=20 |
| --- | --- |
| Median Age at enrollment (IQR) | 15 (15 – 16) |
| Male | 9 (45%) |
| On ART | 20 (100%) |

We began with the stem questions from the TRAQ which included the domains of self-management and self-advocacy with the following questions:

1. Do you fill a prescription if you need to?
2. Do you know what to do if you are having a bad reaction to your medications?
3. Do you take medication correctly and on your own?
4. Do you reorder medication before they run out?
5. Do you call the doctor’s office to make an appointment?
6. Do you follow-up on any referral for tests, check-ups or labs?
7. Do you arrange for your ride to medical appointment?
8. Do you call the doctor about unusual changed in your health?
9. Do you apply for health insurance if you lose your current coverage?
10. Do you know what your health insurance covers?
11. Do you manage your money and budget household expenses?
12. Do you fill out the medical history form, including a list of your allergies?
13. Do you keep a calendar or list of medical and other appointments?
14. Do you make a list of questions before the doctor’s visit?
15. Do you get financial help with school or work?
16. Do you tell the doctor or nurse what you are feeling?
17. Do you answer questions asked by the doctor, nurse or medical staff?
18. Do you help plan or prepare meals?
19. Do you keep your home/room clean?
20. Do you use neighborhood stores or services?

We asked healthcare providers which questions they felt were relevant to their setting and would likely influence transition readiness. When consensus could not be met decisions were based on majority vote.

We then repeated the same process with the TRANSITION-Q using the following stem questions.

1. I answer a doctor’s or nurse’s questions”
2. I help to make decisions about my health
3. I am in charge of taking any medicine that I need
4. I talk to a doctor or nurse when I have health concerns
5. I look for an answer when I have a question about my health
6. I talk about my health condition to people when I need to.
7. I ask the doctor or nurse questions.
8. I speak to the doctor instead of my parents speaking for me.
9. I summarize my medical history when I am asked to.
10. I contact the doctor when I need to.
11. I see the doctor or nurse on my own during an appointment
12. I drop off or pick up my prescriptions when I need medicine.
13. I travel on my own to a doctor’s appointment
14. I book my own doctor’s appointment.

The final list of questions was combined. Feedback was obtained from healthcare providers on appropriate wording for the South African setting and for adolescents. Participants agreed that self-advocacy was an important domain that should be included in the questionnaire. They felt that self-management was better described as health navigation. We then asked if there were additional factors that contributed to transition readiness among adolescents living with HIV in South Africa. There was a consensus among all of the healthcare providers that additional domains of health literacy and disclosure (an adolescent’s awareness of their own HIV status and the ability to communicate that with others) were important contributors. We solicited potential questions from the group to include in the draft questionnaire.

After the draft was obtained we presented the stem questions to focus groups of adolescents for wording clarity. We also asked their input on additional topics to include. The adolescents felt that disclosure (knowledge of their own HIV status) was important prior to transition. We also began with a 4-point Likert scale. The adolescents all agreed to change to a 5-point Likert scale to better encompass the range of potential answers. The final wording was agreed on by the adolescents:

1. No
2. No, but I am learning
3. Yes, a little bit
4. Yes, almost always
5. Yes, always

We also reviewed the instructions with the adolescents for clarity and understanding. The final wording was decided by consensus or majority vote if consensus could not be reached.

The final questions located in Table 3 were based on 3 focus groups from healthcare providers (n=11) and 2 focus groups from adolescents (n=20).
