# Supplementary material for: Development and validation of the HIV adolescent readiness for transition scale (HARTS) in South Africa
Source: J Int AIDS Soc. 2021 Jul 8;24(7):e25767. doi: 10.1002/jia2.25767 (PMC8264413; doi:10.1002/jia2.25767)
Supplement: Supplementary file 2 — Supplement S2. Validation of individual domains using multivariable models assessing viral failure one year after transition to adult care. [file JIA2-24-e25767-s002.docx]

**Supplement 2: Validation of individual domains using multivariable models assessing viral failure one year after transition to adult care.**

**Table S2:** Multivariable logistic regression evaluating self-advocacy and virologic failure one year after transition to adult care for adolescents living with perinatally-acquired HIV at Prince Mshiyeni Hospital, Umlazi, South Africa

| **Covariate** | **AOR** | **P-value** | **95% CI** |
| --- | --- | --- | --- |
| Age at ART initiation (years) | 1.21 | 0.006 | 1.05 – 1.38 |
| Female | 2.25 | 0.024 | 1.11 – 4.55 |
| Alcohol use | 2.87 | 0.011 | 1.27 – 6.47 |
| Documented disclosure | 0.41 | 0.022 | 0.19 – 0.88 |
| Illicit drug use | 3.09 | 0.107 | 0.78 – 12.01 |
| First-line ART | 0.08 | <0.001 | 0.03 – 0.26 |
| **HARTS Domain: Self-advocacy (10 point effect)** | **0.56** | **0.029** | **0.33 – 0.94** |

**Table S3:** Multivariable logistic regression evaluating disclosure and virologic failure one year after transition to adult care for adolescents living with perinatally-acquired HIV at Prince Mshiyeni Hospital, Umlazi, South Africa

| **Covariate** | **AOR** | **P-value** | **95% CI** |
| --- | --- | --- | --- |
| Age at ART initiation (years) | 1.13 | 0.075 | 0.98 – 1.23 |
| Female | 2.06 | 0.069 | 0.94 – 4.48 |
| Alcohol use | 3.80 | 0.005 | 1.48 – 9.72 |
| Documented disclosure | 0.64 | 0.283 | 0.29 – 1.43 |
| Illicit drug use | 5.38 | 0.032 | 1.16 – 25.01 |
| First-line ART | 0.08 | <0.001 | 0.03 – 0.29 |
| **HARTS Domain: Disclosure (10 point effect)** | **0.02** | **0.002** | **0.01 – 0.25** |

**Table S4**: Multivariable logistic regression evaluating health navigation and virologic failure one year after transition to adult care for adolescents living with perinatally-acquired HIV at Prince Mshiyeni Hospital, Umlazi, South Africa

| **Covariate** | **AOR** | **P-value** | **95% CI** |
| --- | --- | --- | --- |
| Age at ART initiation (years) | 1.21 | 0.004 | 1.06 – 1.38 |
| Female | 2.48 | 0.011 | 1.23 – 4.99 |
| Alcohol use | 3.11 | 0.005 | 1.41 – 6.86 |
| Documented Disclosure | 0.40 | 0.019 | 0.18 – 0.86 |
| Illicit drug use | 2.23 | 0.192 | 0.67 – 7.46 |
| First-line ART | 0.09 | <0.001 | 0.03 – 0.27 |
| **HARTS Domain: Health navigation (10 point effect)** | **0.51** | **0.056** | **0.25 – 1.02** |

**Table S5:** Multivariable logistic regression evaluating health literacy and virologic failure one year after transition to adult care for adolescents living with perinatally-acquired HIV at Prince Mshiyeni Hospital, Umlazi, South Africa

| **Covariate** | **AOR** | **P-value** | **95% CI** |
| --- | --- | --- | --- |
| Age at ART initiation (years) | 1.20 | 0.006 | 1.05 – 1.37 |
| Female | 2.27 | 0.021 | 1.13 – 4.57 |
| Alcohol use | 3.08 | 0.007 | 1.37 – 6.93 |
| Documented disclosure | 0.42 | 0.027 | 0.19 – 0.91 |
| Illicit drug use | 2.66 | 0.116 | 0.78 – 9.08 |
| First-line ART | 0.09 | <0.001 | 0.03 – 0.26 |
| **Harts Domain: Health literacy (10 point effect)** | **0.37** | **0.121** | **0.10 – 1.30** |
